# Imbalance of local bone metabolism in inflammatory arthritis and its reversal upon tumor necrosis factor blockade: direct analysis of bone turnover in murine arthritis

**DOI:** 10.1186/ar1872

**Published:** 2005-12-30

**Authors:** Jochen Zwerina, Birgit Tuerk, Kurt Redlich, Josef S Smolen, Georg Schett

**Affiliations:** 1 Division of Rheumatology, Department of Internal Medicine III, Medical University of Vienna, Waehringer Guertel 18-20, A-1090 Vienna, Austria

## Abstract

Chronic arthritis typically leads to loss of periarticular bone, which results from an imbalance between bone formation and bone resorption. Recent research has focused on the role of osteoclastogenesis and bone resorption in arthritis. Bone resorption cannot be observed isolated, however, since it is closely linked to bone formation and altered bone formation may also affect inflammatory bone loss. To simultaneously assess bone resorption and bone formation in inflammatory arthritis, we developed a histological technique that allows visualization of osteoblast function by *in-situ *hybridization for osteocalcin and osteoclast function by histochemistry for tartrate-resistant acid phosphatase. Paw sections from human tumor necrosis factor transgenic mice, which develop an erosive arthritis, were analyzed at three different skeletal sites: subchondral bone erosions, adjacent cortical bone channels, and endosteal regions distant from bone erosions. In subchondral bone erosions, osteoclasts were far more common than osteoblasts. In contrast, cortical bone channels underneath subchondral bone erosions showed an accumulation of osteoclasts but also of functional osteoblasts resembling a status of high bone turnover. In contrast, more distant skeletal sites showed only very low bone turnover with few scattered osteoclasts and osteoblasts. Within subchondral bone erosions, osteoclasts populated the subchondral as well as the inner wall, whereas osteoblasts were almost exclusively found along the cortical surface. Blockade of tumor necrosis factor reversed the negative balance of bone turnover, leading to a reduction of osteoclast numbers and enhanced osteoblast numbers, whereas the blockade of osteoclastogenesis by osteoprotegerin also abrogated the osteoblastic response. These data indicate that bone resorption dominates at skeletal sites close to synovial inflammatory tissue, whereas bone formation is induced at more distant sites attempting to counter-regulate bone resorption.

## Introduction

Rheumatoid arthritis (RA) is one of the most typical examples of a chronic inflammatory process, which leads to profound changes of the skeleton [1]. In fact, RA and other forms of chronic arthritis are major precipitators of bone loss. Structural skeletal damage plays a major role in the outcome of RA patients since functional disability is a result of accumulating changes of the joint architecture [2]. Typically, juxta-articular bone is the skeletal site most exposed to the chronically inflamed RA synovial membrane, which directly invades bone and leads to formation of local erosions. These local bone erosions are characteristic for RA and are part of the diagnostic criteria of the disease [3].

The underlying mechanisms leading to the excessive bone loss in RA are not fully understood, although some key interactions between inflammation and bone, such as the receptor activator of NF-κB ligand (RANKL), have been unraveled during recent years [4-6]. Since bone loss is always the result of a negative net balance of bone formation and resorption, mediators expressed within the synovial tissue are thought to induce a shift from bone formation to bone resorption. For instance, tumor necrosis factor (TNF) enhances osteoclast formation, and thus bone resorption, but it has also negative effects on bone formation since it interferes with differentiation and metabolic activity of osteoblasts [7-9].

The pathological role of altered bone turnover in destructive arthritis is strongly supported by the detection of osteoclasts at sites of local bone erosion. These cells are localized at the interphase of inflammatory tissue and bone, and are found in all animal models of destructive arthritis as well as in human RA [10-13]. Moreover, osteoclast precursors form in the synovial inflammatory tissue, allowing a continuous replenishment of the osteoclast pool necessary to achieve progressive bone damage [5]. Kinetic studies in animal models have also shown that osteoclast formation in the joint is a fast and dynamic process, leading to a rapid attack on juxta-articular bone, the prerequisite of early onset of structural damage [14]. Less is known about the role of osteoblasts in arthritic bone damage. These stromal-derived cells are the natural counterplayers of osteoclasts and might serve as a potential repair mechanism. In fact, osteoblasts are found within the arthritic bone erosions of animal models of arthritis as well as human RA [10]. In addition, stimulation of osteoblasts by parathyroid hormone has proven a repair process in experimental arthritis [15].

The relation of the metabolic activities of osteoclasts, evoking bone erosion, and of osteoblasts, mediating repair, is crucial for understanding the process of skeletal damage in arthritis. However, techniques that allow assessing the activity of both cell types simultaneously have not so far been employed. Here, using a new technique that facilitates simultaneous detection of metabolically active osteoclasts and osteoblasts in one histological section, and thus enables a direct investigation of bone turnover in arthritis, we have analyzed the role of osteoblasts relative to osteoclasts in experimental arthritis. As the experimental model for destructive arthritis we chose mice that are transgenic for human tumor necrosis factor (hTNFtg), since these mice develop a destructive arthritis closely mimicking human RA [16] and their disease is based on overexpression of TNF, which is centrally involved in joint inflammation and arthritic bone loss.

## Materials and methods

### Animals and treatments

The heterozygous hTNFtg mice (strain Tg197; genetic background, C57BL/6) have been described previously [16]. These mice develop a chronic inflammatory and destructive polyarthritis within 4–6 weeks after birth. In the present study, a total of 35 mice were examined in two independent experiments comprising 17 mice and 18 mice, respectively. The mice were divided into two groups in a 5:1:1 ratio and were treated according to the following protocol: group 1 received PBS (negative control); group 2 received a chimeric monoclonal anti-TNF antibody (infliximab, kindly provided by Centocor Pharmaceuticals, Leiden, The Netherlands) intraperitoneally at a dose of 10 mg/kg three times per week; and group 3 received osteoprotegerin (OPG) (an OPG-Fc fusion protein; Amgen, Thousand Oaks, CA, USA) intraperitoneally at a dose of 10 mg/kg three times a week. Therapy was started at the onset of symptoms (week 5) and lasted for 6 weeks. At the end of the study, mice were sacrificed by cervical dislocation, blood was drawn by heart puncture and the hind paws were obtained for histological evaluation. The local ethics committee approved all animal procedures.

### Preparation of specimens for histological evaluation of bone erosions

Right hind paws were fixed in 4% paraformaldehyde overnight and were decalcified in 14% ethylenediamine tetraacetic acid (Sigma, St Louis, MO, USA) at 4°C (pH adjusted to 7.2 by addition of ammonium hydroxide; Sigma) until the bones were pliable. Serial paraffin sections (2 µm) were stained with H & E for evaluation of synovial inflammation and bone erosions.

### Tartrate-resistant acidic phosphatase-osteocalcin double labeling

Double labeling of osteoclasts and osteoblasts was performed by combining tartrate-resistant acidic phosphatase (TRAP) labeling for detection of osteoclasts and osteocalcin *in-situ *hybridization for detection of osteoblasts. Sections were first stained with TRAP (leukocyte acid phosphatase kit; Sigma) as described previously [5]. The sections were subsequently prepared for *in-situ *hybridization by post-fixating paw sections with 4% paraformaldehyde for 10 minutes at room temperature, rinsing with 0.1% Tween in PBS and digesting with proteinase K (1 µg/ml; Roche, Mannheim, Germany). After rinsing again with 0.1% Tween in PBS, another post-fixation step with 4% paraformaldehyde for 10 minutes at room temperature was carried out, followed by acetylation in acetic anhydride (2.5 µl/ml in triethanolamine buffer) for 15 minutes at room temperature and air drying for 30 minutes at 37°C.

The cDNA probe for the osteocalcin *in-situ *hybridization was kindly provided by Christine Hartmann (Research Institute of Molecular Pathology, Vienna, Austria). The osteocalcin probe consisted of a 600 bp insert cleaved with *Not*I and transcribed with T3 RNA polymerase to generate antisense probes using digoxygenin labeling (DIG RNA labeling kit; Roche). The digoxygenin-labeled osteocalcin probe was diluted 1:100 in hybridization solution (10 mM Tris, pH 7.5, 600 mM NaCl, 1 mM ethylenediamine tetraacetic acid, 0.25% SDS, 10% dextrane sulfate, 1 × Denhardt's solution, 200 µg/ml yeast tRNA, 50% formamide) and heated to 85°C for three minutes. The sections were then incubated with the probe, covered with coverslips and stored overnight in a humified chamber at 65°C. After removal of the coverslips, the slides were rinsed with 1 × SSC/50% formamide for 30 minutes at 65°C, followed by digestion of single-stranded RNA with RNase A (20 µg/ml in TNE buffer; Roche) for 10 minutes at 37°C and washing steps in TNE buffer (10 mM Tris, 500 mM NaCl, 1 mM ethylenediamine tetraacetic acid) for 10 minutes at 37°C and in 2 × SSC and 0.2 × SSC for 20 minutes each at 65°C.

Detection of the probe was performed by incubating the slide with MABT buffer (100 mM maleic acid, 150 mM NaCl, 0.1% Tween 20, pH 7.5) for 5 minutes at room temperature before blocking with 20% heat-inactivated sheep serum in MABT buffer for 1 hour. The sections were then incubated with alkaline phosphatase-labeled anti-digoxygenin antibody (1:2000; Roche) in 2% heat-inactivated sheep serum/MABT buffer at 4°C in a humified chamber. For the color reaction, nitroblue tetrazolium (0.25 µg/ml) and 5-bromo-4-chloro-3-indolyl phosphate (0.125 µg/ml) dissolved in NTMT buffer (100 mM NaCl, 100 mM Tris, pH 9.5, 50 mM MgCl_2_, 0.1% Tween 20) was applied at room temperature overnight.

### Histomorphometry

All analyses were performed using a microscope (Zeiss Axioskop 2; Zeiss, Marburg, Germany) equipped with a digital camera and an image analysis system (Osteomeasure; OsteoMetrics, Decatur, GA, USA) as described previously [17]. The area of bone erosion was quantified in all tarsal joints of all hind paws in H & E-stained axial sections of hind paws. Osteoclasts (three or more nuclei, purple color) and osteoblasts (dark blue color) were identified in the TRAP-osteocalcin double-stained slides. Quantification of these cells was separately carried out in three different compartments of the juxta-articular bone: the subchondral bone erosion, the subchondral bone channels (also termed Haversian channels) within cortical bone adjacent to the inflamed joints, and the endosteal surface at distant sites from bone erosion. Moreover, within synovial bone erosions, the distribution of osteoclasts and osteoblasts was assessed at: the surface adjacent to the articular cartilage (subchondral surface), the inner frontier of erosion (inner surface), and the surface adjacent to the cortical bone (cortical surface). Two different histomorphometrical analyses were performed: the number of osteoclasts as well as osteoblasts per bone perimeter (cell numbers per millimeter of bone surface), and the amount of bone surface covered by osteoclasts and osteoblasts (percentage). All analyses were carried out by a single investigator in blinded fashion.

### Statistical analysis

Data are presented as the mean ± standard error. Group mean values were compared by the two-tailed Student *t *test. The non-parametric Spearman test was used for correlation analysis,.

## Results

### Synovial inflammation affects juxta-articular bone at different compartments

**Figure 1:**
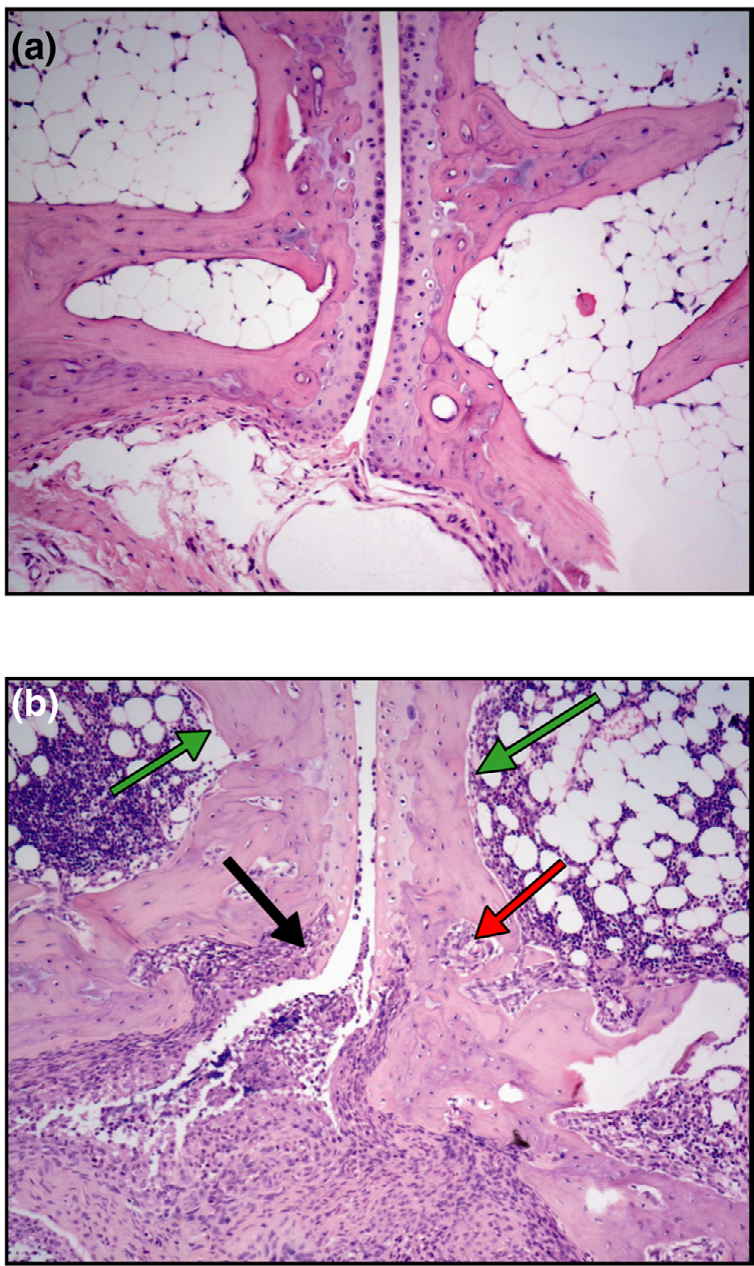
Synovial inflammation affects different compartments of juxta-articular bone. H & E-stained sections from the tarsal region of healthy 12-week-old (a) wild-type mice and (b) mice transgenic for human tumor necrosis factor (hTNFtg). The hTNFtg mice develop extensive synovial inflammation, cartilage damage and bone erosions. Three bone compartments affected by synovitis were analyzed in arthritic mice: the subchondral bone adjacent to the synovial inflammatory tissue (black arrow), the cortical bone channels in close vicinity of the inflamed areas (red arrow), and the endosteal bone surface distant from the inflammatory tissue (green arrows).

Based on the knowledge of arthritic bone loss in histological studies of animal models of arthritis and the radiographic appearance of arthritic bone erosions in human disease, we defined three different compartments of periarticular bone, where a direct *in situ *analysis of bone turnover would be of interest (Figure 1). The first is the cortical bone directly adjacent to articular cartilage at the cartilage-pannus junction (Figure 1b, black arrow), which is most directly affected by synovial inflammatory tissue leading to formation of subchondral bone erosions. Another compartment is the area of cortical bone channels, also termed Haversian channels (Figure 1b, red arrow), which is somewhat more distant, albeit still in close vicinity to the synovial inflammatory tissue. The final compartment is the endosteal surface of bone at sites distant from inflammatory tissue (Figure 1b, green arrows). We analyzed bone turnover in these compartments in hTNFtg mice, which develop a chronic destructive arthritis similar to RA. In this model, tarsal joints are among the first affected by arthritis, which is characterized by a hyperplastic synovial membrane invading the subchondral bone (Figure 1).

### Compartments with different patterns of bone turnover in inflammatory arthritis

**Figure 2:**
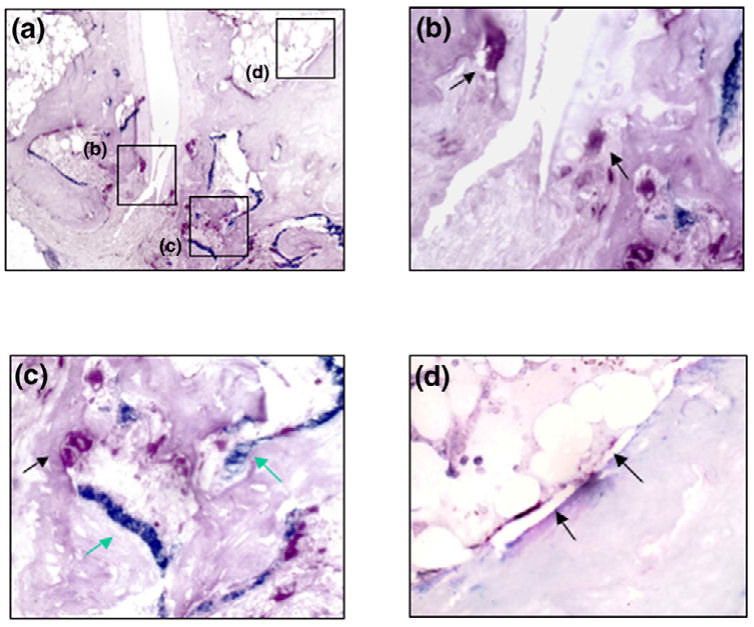
Double labeling for osteocalcin and tartrate-resistant acidic phosphatase for visualization of osteoblasts and osteoclasts. Paraffin-embedded sections of hind paws from arthritic mice transgenic for human tumor necrosis factor (hTNFtg) were stained by TRAP to determine osteoclasts (black arrows; purple-colored cells with three or more nuclei). In the same sections, osteoblasts were visualized by *in-situ *hybridization for osteocalcin (green arrows; deep blue-stained cells). (a) Overview of a representative section, original magnification × 100. (b) Close-up view of the compartment of subchondral bone erosion, original magnification × 400. (c) Cortical bone channels underneath subchondral bone erosions, original magnification × 400. (d) Inflamed endosteal bone surface distant from bone erosion, original magnification × 400.

To assess bone turnover in the various juxta-articular bone compartments during arthritis, we developed a technique to simultaneously assess the metabolic activity of osteoclasts and osteoblasts *in situ *by detection of TRAP for osteoclast activity and of osteocalcin for osteoblast activity. Representative images of the different skeletal regions showing osteoclasts and osteoblasts simultaneously are depicted in Figure 2.

**Figure 3:**
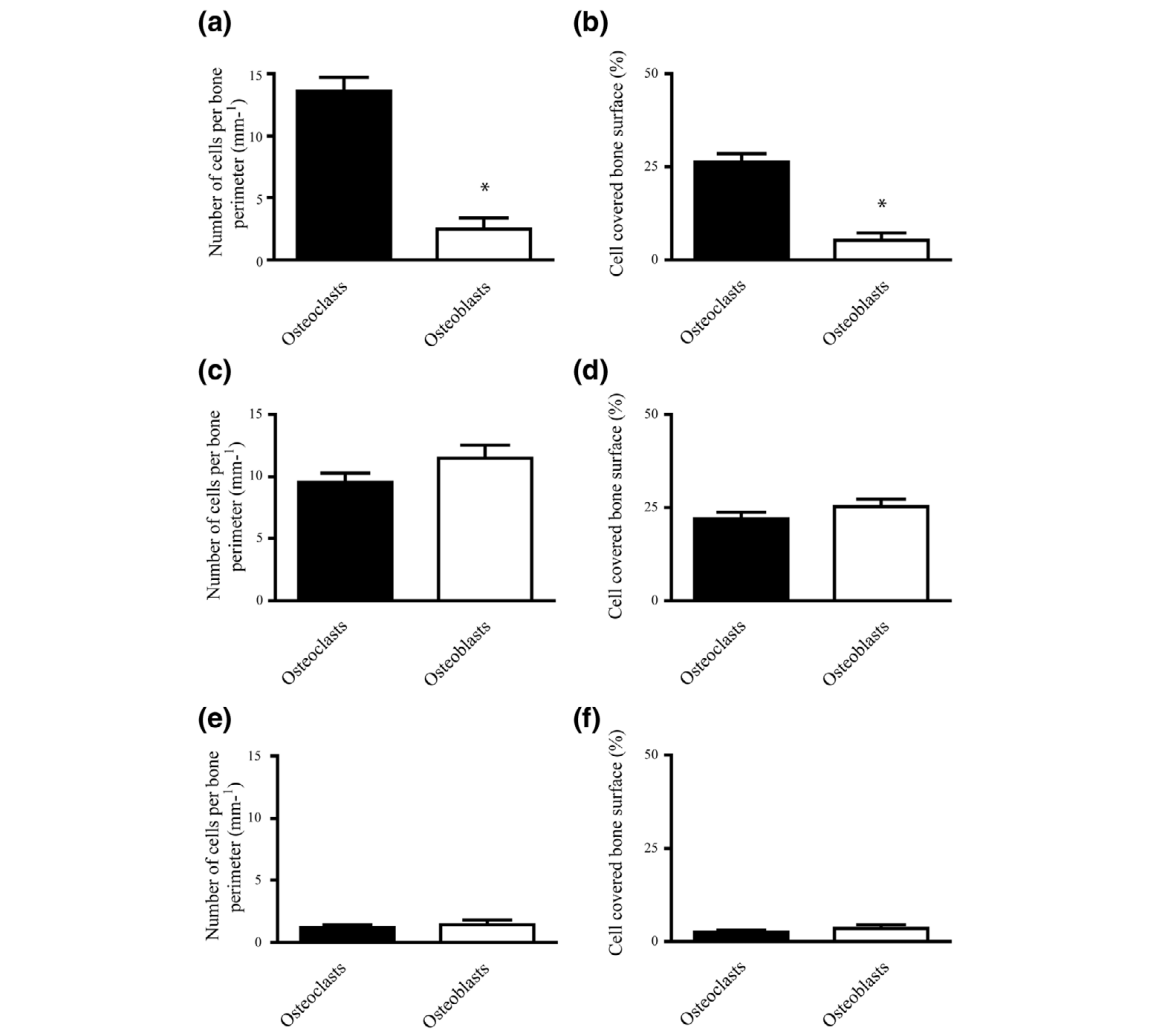
Arthritis induces different patterns of bone turnover in the various compartments of juxta-articular bone. Untreated 12-week-old arthritic mice transgenic for human tumor necrosis factor were evaluated for the numbers (a, c, e) and density (b, d, f) of functional osteoclasts by labeling tartrate-resistant phosphatase and osteoblasts by *in-situ *hybridization for osteocalcin. Three compartments were analyzed by histomorphometry: (a, b) subchondral bone, (c, d) cortical bone channels and (e, f) the endosteal bone surface distant from inflamed areas. Results are presented as numbers of cells per mm bone surface (a, c, e) and the fraction (%) of bone surface covered by cells (b, d, f). *Statistically significant difference, *P *< 0.05.

All hTNFtg mice showed accumulation of osteoclasts and osteoblasts in the inflamed joint region, whereas such cells were virtually absent in wild-type mice (data not shown). Within subchondral bone erosions, the number of osteoclasts significantly (*P *< 0.01) exceeded osteoblasts (mean ± standard error 13.6 ± 1.1 osteoclasts versus 2.5 ± 0.9 osteoblasts per mm bone surface; Figure 3a). Also, a fivefold larger bone surface was covered by osteoclasts (26.0 ± 2.1%) than by osteoblasts (5.4 ± 1.9%; Figure 3b). This indicates high bone turnover within subchondral bone with an excess in bone resorption. In cortical bone channels, however, we observed an equal distribution of osteoclasts and osteoblasts (9.5 ± 0.8 osteoclasts and 11.5 ± 1.1 osteoblasts per mm bone surface), both covering approximately 25% of the bone surface, indicating high but balanced bone turnover (Figure 3c,d,). In contrast, the endosteal surface distant from bone erosions contained only few bone cells. Mean numbers of 1.2 (± 0.3) osteoclasts and 1.4 (± 0.4) osteoblasts per mm bone surface were detected, attributing to only 2.5% and 3.5% bone surface covered by osteoclasts and osteoblasts, respectively (Figure 3e,f). This indicates a low bone turnover at skeletal sites distant from bone erosions in inflammatory arthritis.

### Localization of osteoclasts and osteoblasts in local bone erosions of TNF transgenic mice

Next, we determined the distribution of osteoclasts and osteoblasts in the primary skeletal lesion of arthritis: subchondral bone erosion. Three compartments were defined within these lesions: the surface adjacent to the articular cartilage (subchondral surface), the inner frontier of erosion (inner surface), and the surface adjacent to cortical bone (cortical surface).

**Figure 4:**
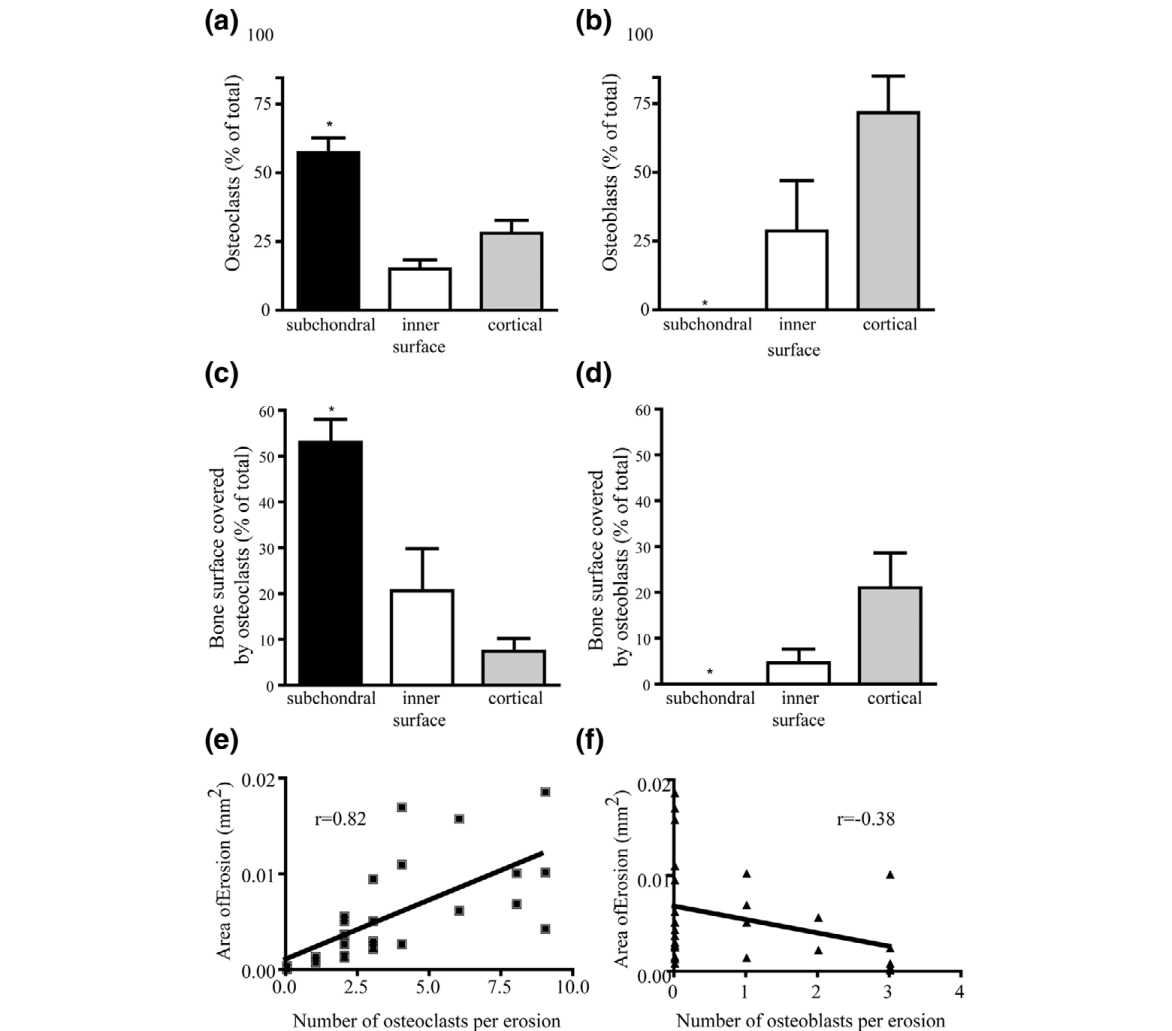
Distribution of osteoclasts and osteoblasts within local bone erosions. Paraffin-embedded sections of hindpaws of mice transgenic for human tumor necrosis factor were simultaneously stained for (a, c) osteoclasts and (b, d) osteoblasts. The distribution and density of osteoclasts and osteoblasts were assessed in three different compartments within bone erosions: the surface adjacent to the articular cartilage (subchondral surface), the inner frontier of erosion (inner surface), and the surface adjacent to cortical bone (cortical surface). The size of local bone erosions was correlated to the numbers of (e) osteoclasts and (f) osteoblasts within these lesions. Spearman's correlation coefficients are given. The numbers of osteoclasts correlated positively (*r *= 0.82, *P *< 0.0001) with the area of bone erosion, whereas the numbers of osteoblasts correlated negatively (*r *= -0.38, *P *< 0.05).

Histomorphometrical analysis revealed that more than one-half of the osteoclasts (57%) were located at the subchondral bone surface, whereas fewer osteoclasts were found at the cortical bone surface (28%) and inner bone surface (15%), respectively (Figure 4a). The distribution of osteoblasts in subchondral bone erosions was inverse: most osteoblasts were found at the cortical bone surface (71%), fewer at the inner surface (29%), and no osteoblasts were seen at the subchondral surface of the erosions (Figure 4b). Similar results were also obtained when measuring the density of osteoclasts and osteoblasts at these different compartments: 53% of the subchondral bone surface was covered by osteoclasts, whereas none was covered by osteoblasts. At the inner bone surface, 21% and 5% bone was covered by osteoclasts and osteoblasts, respectively. In contrast, osteoblasts at the cortical bone surface covered a significantly greater amount of bone surface (21%) than osteoclasts (7%) (Figure 4c,d). Hence, osteoclasts are more frequent at the subchondral bone surface, whereas osteoblasts are most abundant at the cortical surface of bone erosions.

We next examined whether the size of subchondral bone erosions was linked to the amount of osteoclasts and osteoblasts within these lesions. A strong positive correlation (*r *= 0.82; *P *< 0.001) was found when correlating the number of osteoclasts with bone erosion size (Figure 4e). Interestingly, the number of osteoblasts at the site of bone erosion correlated inversely with the area of bone erosion (*r *= -0.38, *P *< 0.05; Figure 4f).

### Reversed local bone turnover upon anti-TNF treatment in erosive arthritis

We analyzed the effects of TNF blockade on local bone turnover. TNF blockade was initiated at the stage of early arthritis and was continued for six weeks. We then determined the extent of local bone damage and quantified the area of subchondral bone erosions. Whereas untreated TNF transgenic mice exhibited extensive bone erosions (mean ± standard error, 6330 ± 1060 µm^2^), anti-TNF treatment strongly reduced the area of bone erosions to 450 ± 170 µm^2 ^(*P *< 0.001).

Moreover, the microenvironment in the residual subchondral bone erosions showed a dramatic change compared with untreated mice. As shown in Table 1, the number of osteoclasts was dramatically reduced to a mean of 1.9 ± 1.9 per bone perimeter (-86%), covering only 3 ± 3% of the bone surface (-88% when compared with untreated animals, as shown in Figure 3). In contrast, the number of osteoblasts was strikingly increased to a mean of 47.9 ± 13.4 cells per bone perimeter (+1600%) resulting in 37% bone surface coverage by osteoblasts (+685%). A similar picture was observed when analyzing cortical bone channels. Osteoclasts decreased to a number of 8.9 ± 1.9 (-7%), covering 9% of the bone surface (-64%). At the same time, osteoblasts had expanded (26 ± 7.4 osteoblasts per mm bone perimeter [+226%]), reflecting 43% covered bone surface (+172%). At endosteal sites distant from bone erosions, the numbers of osteoclasts per bone perimeter remained unchanged (1.6 ± 0.7), covering 0.6% of the bone surface. The numbers of osteoblasts, however, increased to 11.2 ± 5.1 per bone perimeter, covering a mean of 4% of the endosteal bone surface.

TNF blockade therefore completely reverses increased bone resorption and leads to a dramatic increase in osteoblast numbers, indicating high bone turnover with a positive net balance in anti-TNF-treated arthritic mice.

### Blockade of osteoclastogenesis abolishes osteoblast activity, indicating the osteoblastic response is dependent on bone erosion

To determine whether specific inhibition of osteoclastogenesis without affecting synovial inflammation influences the anabolic response in bone of arthritic mice, we treated 6-week-old hTNFtg mice three times per week for 6 weeks with OPG. This treatment did not significantly inhibit joint inflammation (data not shown) but did strongly diminish local bone erosions by 90% (mean ± standard error, 6330 ± 1060 µm^2 ^for untreated mice versus 618 ± 860 µm^2 ^for OPG-treated mice) compared with TNF blockade. Analysis of osteoclasts and osteoblasts within the remaining bone erosions not only showed the anticipated dramatic reduction of osteoclasts in bone erosions (0.6 ± 0.4 osteoclasts per mm, -96%), but also a complete absence of osteoblasts (Table 1). A similar picture emerged when cortical bone channels were analyzed: almost no osteoclasts (0.4 ± 0.4 per mm bone surface) and almost no osteoblasts (0.2 ± 0.2 per mm bone surface) were detectable, attributing to less than 1% of a bone surface coverage by bone active cells. Similarly, no bone cells were seen at the endosteal bone surface distant from bone erosions.

Selective blockade of osteoclasts in arthritic mice is therefore accompanied by a strong reduction of osteoblasts, suggesting that accumulation of osteoblasts is a consequence of arthritic bone resorption and not of synovial inflammation *per se*.

## Discussion

Skeletal turnover is a complex physiological process, which requires bone resorption mediated by osteoclasts as well as bone formation exerted by osteoblasts. Chronic inflammation can lead to sustained alterations of bone turnover, as typically seen in juxta-articular and periarticular damage of inflamed joints in RA. TNF is a proinflammatory cytokine of major importance in chronic inflammatory arthritis and also exerts profound effects on bone: TNF can induce osteoclast formation, whereas it has negative effects on bone formation. Its unfavorable skeletal effects on bone make TNF an ideal candidate for linking inflammation and bone loss. Indeed, the therapeutic blockade of TNF is highly effective in preventing and/or reducing skeletal damage in conditions of chronic arthritis [18].

**Figure 5:**
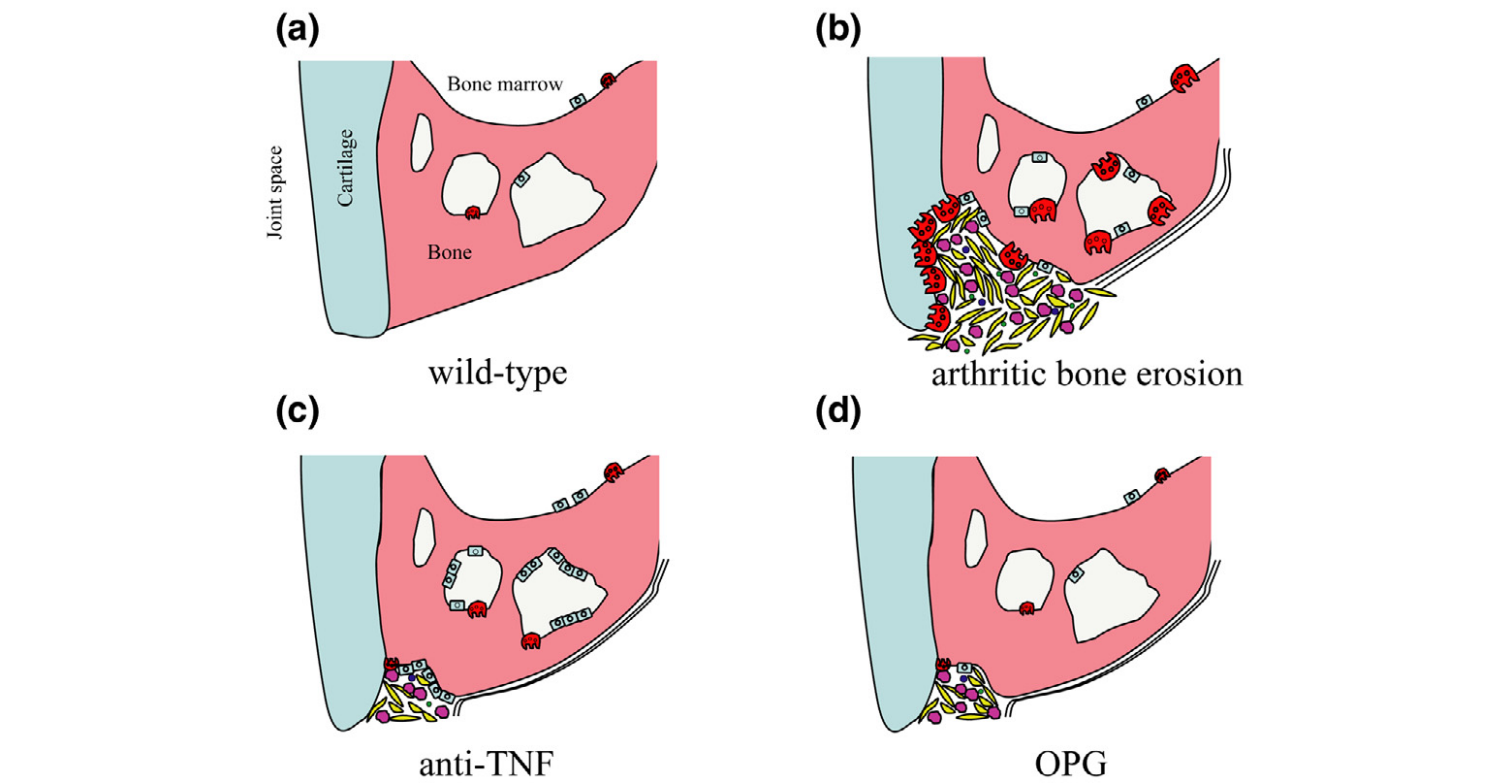
Overview on local bone turnover in tumor necrosis factor transgenic mice. (a) Schematic overview of a healthy joint with only scarce osteoblasts (cubic-shaped blue cells) and osteoclasts (multinucleated red cells) in cortical bone channels and the endosteal bone surface. (b) In arthritis, synovial inflammatory tissue comprised of synovial fibroblasts (yellow spindle-shaped cells), macrophages (purple cells), T cells (green cells), B cells (dark blue cells) and other cells invade the subchondral bone and trigger massive local osteoclastogenesis and – as a reactive process – osteoblastogenesis. Also, osteoclast and osteoblast accumulation occurs in cortical bone channels but not at the endosteal bone surface distant from synovitis. (c) TNF blockade efficiently diminishes bone erosion and suppresses osteoclastogenesis in subchondral bone erosions and cortical bone channels. In contrast, enhanced osteoblast activity can be detected in both regions, suggesting an increased reparative response when TNF is blocked. (d) Receptor activator of NF-κB ligand blockade inhibits bone erosion but not synovitis. Osteoclastogenesis is efficiently blocked in all compartments but, in contrast to anti-TNF, osteoblasts are also massively reduced. OPG, osteoprotegerin.

In the current work, while developing a technique allowing direct visualization and quantitative assessment of metabolically active bone cells to simultaneously analyze functional osteoclasts and osteoblasts representing areas of bone loss and bone formation, respectively, we evaluated juxta-articular and periarticular bone turnover in the context of chronic arthritis. Using a model of TNF overexpression, these analyses revealed several interesting insights into arthritic bone loss, as illustrated in Figure 5.

First, bone turnover was massively increased at sites of joint inflammation. Arthritis in hTNFtg mice involves peripheral joints, such as tarsal, carpal and digital joints, largely reflecting the distribution of inflammation found in human disease. Small peripheral bones predominantly consist of cortical bone, which has a slow turnover compared with trabecular bone. In physiological conditions, therefore, only few osteoclasts and osteoblasts are found at these peripheral skeletal sites. In arthritis, however, accumulation of osteoclasts but also of osteoblasts is found at sites where these cells are normally absent or are only scarcely present [5]. On the basis of these data, the hypothesis that the inflammatory events in RA primarily lead to activation of osteoclasts and bone resorption by means of TNF and RANKL, but do not induce the osteoblast compartment and consequent bone formation, needs to be revisited.

While the negative effects of TNF on bone formation are well documented [8], synovial inflammation in fact appears to also provoke an osteoblastic response, which may be seen as an attempt at bone repair. In fact, the observed accumulation of osteoblasts may be considered as a skeletal response to TNF-mediated bone loss [19]. Osteoclasts and osteoblasts are in close vicinity to each other, suggesting a dynamic interplay between these cells, and consequently between bone resorption and formation at these skeletal sites. This hypothesis is supported by the observation of the metabolically active state of both cell types as observed by the expression of TRAP, a key enzyme for osteoclasts [20], as well as osteocalcin, a specific extracellular matrix molecule produced by osteoblasts [21]. Moreover, accumulation of both cell types was linked to synovial inflammatory tissue, since the number of active osteoblasts and osteoclasts decreased dramatically with growing distance from the inflamed joint, suggesting that mediators originating from inflammatory tissue influence activation of bone turnover by paracrine mechanisms.

A second insight is that the net effect on bone was highly dependent on the proximity to the inflamed synovial membrane. The area closest to inflammatory tissue is the subchondral bone of the junction zone, where the synovial membrane inserts into the cortical bone surface. This site faces rapid resorption, supported by the massive accumulation of osteoclasts, which by far outweighs the number of active osteoblasts in these lesions. The result is a negative net balance of bone formation compared with resorption and progressive local bone erosions. It also reflects the direct effects of a chronic exposure of adjacent bone to proinflammatory cytokines from the synovial membrane and cells (osteoclasts) generated in the synovial membrane. In deeper skeletal layers, however, the osteoblastic response tends to outweigh bone resorption, as evident by similar numbers of osteoblasts and osteoclasts in cortical bone channels underneath erosions. These areas are characterized by high bone turnover with abundance of metabolically active osteoclasts and osteoblasts.

These differences between the compartments suggest that the inflammatory tissue directly drives osteoclastogenesis, which is a well-known process and involves the expression of TNF by various cells types within inflammatory tissue and of RANKL by T cells and synovial fibroblasts [22-25]. On the other hand, these data show that the skeletal response to increased bone resorption emerges from deeper layers of the cortical bone and involves accumulation of osteoblasts synthesizing new bone matrix. A similar state is even found within subchondral bone erosions: although in these lesions only a minority of bone-active cells are osteoblasts, they are almost exclusively found in the deeper cortical areas surrounding the bone erosion but are absent in the superficial area adjacent to articular cartilage. This suggests that the catabolic signal for bone is strongly associated with inflammatory tissue, whereas the anabolic response comes from deeper layers of cortical bone.

Third, hypothesizing that the anabolic skeletal response is an indirect effect, which depends on bone resorption induced by synovial inflammatory tissue, we aimed to shut down osteoclastogenesis specifically by interfering with RANKL-receptor activator of NF-κB interaction. Blockade of RANKL has proven to effectively prevent arthritic bone erosion without interfering with synovial inflammation [11,26-29]. Interestingly, the anabolic response was almost completely abolished when osteoclast generation was blocked by OPG. Previous studies assessing the systemic bone changes in hTNFtg mice have shown that OPG treatment not only blocks bone resorption, but also downregulates bone formation [19]. This is also in line with the effect of RANKL blockers in human clinical trials. The molecular background of the inhibitory effect of RANKL blockers in bone formation is poorly understood. It cannot be ruled out that osteoblastogenesis is dependent on RANKL; however, such an effect has only been described for glutathione S-transferase fusion RANKL (GST-RANKL), not for native RANKL [30].

More generally, however, these observations favor the concept that the generation of viable osteoclasts is necessary for optimal bone anabolic responses [31]. Such a hypothesis of coupling stress is also supported by recent studies, which suggest that acidification of the resorption compartment is an important stimulus for the influx of bone-forming cells into resorption sites [32]. An alternative explanation for osteoblastogenesis next to inflamed joints is that synovial inflammation is directly responsible for the anabolic response of cortical bone. However, several points of evidence contradict such a mechanism: proinflammatory mediators, such as TNF, are negative regulators and not positive regulators of osteoblasts; osteoclasts but not osteoblasts dominate the areas close to synovial inflammatory tissue; accumulation of osteoblasts is always linked to the presence of osteoclasts and is not seen as an isolated process upon emergence of arthritis; and selective blockade of bone resorption by RANKL blockade does not interfere with synovitis, but does block the anabolic response. This clearly indicates that the accumulation of metabolically active osteoblasts at sites of inflammatory bone erosions is not directly based on joint inflammation *per se*, but is rather a consequence of osteoclastogenesis and active bone resorption.

Fourth, from a therapeutic standpoint the reversibility of the pathophysiology of destructive arthritis is a key point of interest. Since in this model TNF is not only responsible for inflammatory arthritis but also for alteration bone turnover [19], removal of this essential trigger might also reverse dysregulation of bone turnover. Indeed, treatment with a TNF blocker completely reversed bone turnover by blocking osteoclasts and fostering osteoblasts. In subchondral sites of bone erosion, where osteoclasts dominated highly, TNF blockade completely switched negative bone turnover to an anabolic phenotype, leading to an excess amount of osteocalcin-synthesizing osteoblasts compared with osteoclasts. This was partially achieved by an effective inhibition of osteoclastogenesis, since TNF is not only a direct activator of osteoclasts but also induces expression of RANKL [9]. However, it was also based on an increase of osteoblasts, which illustrates the mentioned negative role of TNF on bone formation.

Whether these effects of TNF blockade on local bone metabolism are long-term effects is currently unknown, since the duration of treatment, although comparatively long for a model of inflammatory arthritis, is not designed to assess potential long-term effects. Another potential limitation is that no dose titration of skeletal effects of TNF and/or RANKL blockers has been accomplished, since blockade of TNF and RANKL was used as a proof-of-concept study on the role of TNF and osteoclasts in local skeletal remodeling in this experimental model.

## Conclusion

This investigation extends current insights into bone loss in the context of inflammatory arthritis. Bone erosion is not a purely resorptive process, but rather harbors complex interactions of bone resorption and formation throughout the entire cortical bone. Induction of bone formation in deeper layers of cortical bone can be regarded as an attempt to auto-repair, which limits but does not reverse skeletal damage. This process depends on the potential of synovial inflammatory tissue to generate osteoclasts and to resorb bone, but is not a consequence of synovial inflammation *per se*. Most importantly, distorted bone metabolism is reversible when the initial trigger, primarily TNF, is neutralized – suggesting that TNF blockade might not only block bone resorption but also pave the path for effective repair of skeletal damage.

## Abbreviations

bp = base pair; H & E = haematoxylin and eosin; hTNFtg = transgenic for human tumor necrosis factor; M-CSF = macrophage stimulating factor; NF = nuclear factor; OPG = osteoprotegerin; PBS = phosphate buffered saline; RA = rheumatoid arthritis; RANKL = receptor activator of NF-κB ligand; TNF = tumor necrosis factor; TRAP = tartrate-resistant acidic phosphatase.

## Competing interests

The authors declare that they have no competing interests.

## Authors' contributions

JZ carried out breeding of the mice, performed histological analyses and drafted the manuscript. BT carried out histological analyses. KR and JSS participated in the design of the study. GS conceived the study, and participated in its design and coordination. All authors read and approved the final manuscript.

## Figures and Tables

**Table 1:** TNF and RANKL blockade differently alter local bone turnover in hTNF-transgenic mice

Mice	OcPm/BPm (%)	ObPm/BPm (%)	NOc/BPm (cells/mm)	NOb/BPm (cells/mm)
Untreated hTNFtg				
Subchondral bone erosion	26 ± 2	5 ± 2	13.5 ± 1.1	2.5 ± 0.8
Cortical bone channels	22 ± 1	25 ± 2	9.5 ± 0.7	11.4 ± 1.0
Endosteal bone surface	1 ± 1	4 ± 1	1.2 ± 0.2	1.4 ± 0.3
OPG-treated hTNFtg				
Subchondral bone erosion	1 ± 1	0	0.6 ± 0.4	0
Cortical bone channels	1 ± 1	1 ± 1	0.4 ± 0.4	0.2 ± 0.2
Endosteal bone surface	0	0	0	0
Anti-TNF treated hTNFtg				
Subchondral bone erosion	3 ± 3	36 ± 5	4.1 ± 1.2	48.3 ± 11.4
Cortical bone channels	9 ± 2	43 ± 10	8.7 ± 3.3	26.1 ± 7,2
Endosteal bone surface	1 ± 1	4 ± 1	2.5 ± 1.2	10.9 ± 5.4

Results presented as the mean ± standard error of the mean. Paw sections of transgenic for human tumor necrosis factor (hTNFtg) mice treated for six weeks with a neutralizing antibody against TNF (anti-TNF) or osteoprotegerin (OPG) were stained for functional osteoclasts and osteoblasts. The fraction of bone surface covered by osteoclasts and osteoblasts (OcPm/BPm, ObPm/BPm) and the numbers of osteoclasts and osteoblasts per bone perimeter (NOc/BPm, NObPm) were assessed and allocated to three skeletal compartments.
